# Successful nonsurgical therapy of a diabetic foot osteomyelitis in a patient with peripheral artery disease with almost complete radiological restoration

**DOI:** 10.1186/s13104-018-3694-x

**Published:** 2018-08-13

**Authors:** C. V. Loupa, E. Meimeti, E. Voyatzoglou, A. Donou, E. Koutsantoniou, S. Lafoyanni

**Affiliations:** 1 Demetrios Voyatzoglou Diabetic Foot Clinic, Amalia Fleming Hospital Unit, Athens, Greece; 2 Radiology Department, Amalia Fleming Hospital Unit, Athens, Greece

**Keywords:** Osteomyelitis, Peripheral artery disease, Diabetes mellitus, Diabetic foot infection, Cellulitis, Angioplasty, Surgical debridement, Nonsurgical therapy

## Abstract

**Background:**

Diabetic foot ulcer (DFU) is a common complication in patients with diabetes mellitus (DM) and can consequently lead to soft tissue infection and osteomyelitis.

**Case presentation:**

We present a case of a 68-year-old man with a history of Type 2 DM and symptomatic peripheral artery disease, referred to our hospital due to an infected lower extremity DFU. Cultures revealed methicillin-resistant *Staphylococcus aureus* and *Stenotrophomonas maltophilia*. There was a significant increase of inflammatory marker levels and plain X-rays revealed osteomyelitis. He underwent lower extremity angioplasty for the restoration of the blood flow. He received targeted intravenous antibiotic therapy for 2 weeks and continued ciprofloxacin along with clindamycin per os for 10 more weeks as outpatient.

**Conclusion:**

As a result, the patient presented almost complete healing of his DFU, reconstruction of osteomyelitis defects in X-ray and complete restoration of his foot functionality only 4 months after the end of the treatment. This case demonstrates a DFU complicated by osteomyelitis which resolved medically and nonsurgically, with the exception of surgical restoration of the blood flow.

## Background

Diabetic foot ulcer (DFU) is a chronic and common complication of diabetes mellitus (DM), with a yearly incidence range from 2 to 4% in developed countries [1]. Osteomyelitis is one of the most common expression of diabetic foot infection, being present approximately in present in 10–15% of moderate and in 50% of severe infectious process [2]. Osteomyelitis occurs after a soft tissue infection in the DFU area that spreads into the bone, involving the cortex first and then the marrow. DFU and consequently osteomyelitis has been established as important risk factors for minor or major lower-extremity amputation [3].

On the other hand, peripheral artery disease (PAD) often coexists in patients with DM and DFUs. PAD and infection influence the evolution of DFUs, increasing the risk of non-healing and is associated with a poor outcome [3]. The presence of clinically significant foot ischaemia makes both diagnosis and treatment of infection considerably more difficult. It is generally recommended in the case of diabetic foot osteomyelitis and PAD to perform revascularization to restore a proper blood flow to the infected limb.

Accurate diagnosis of bone infection can be difficult, but is essential to ensure appropriate treatment. Of course, proper antibiotic therapy plays the most significant role in the treatment of diabetic foot (DF) osteomyelitis. Aerobic Gram positive cocci (such as *Staphylococcus aureus*, *S. epidermidis*, streptococci), and especially *S. aureus,* are the most commonly detected bacteria in DF infections, followed by Gram negative rods (such as Enterobacteriaceae). Methicillin-resistant *Staphylococcus aureus* (MRSA) plays an increasing role in DF infections nowadays [3]. MRSA is often isolated from diabetic patients who have recently received antibiotic therapy, have been previously hospitalized or reside in a chronic care facility.

Therefore, it is very important to act rapidly and effectively in order to avoid amputation.

Although traditionally osteomyelitis has been surgically treated, nowadays there is a tendency towards medical therapy alone [3, 4]. In the Infectious Diseases Society (IDSA) guidelines [3], there is a list of osteomyelitis cases that a nonsurgical treatment can be tried.

We present a case of successful treatment of a DF infection complicated by osteomyelitis. Patient was treated effectively nonsurgically, with the use of targeted, initially parenteral and after that per os antibiotics. The treatment was successful, and the X-ray image was almost entirely restored.

## Case presentation

**Fig. 1:**
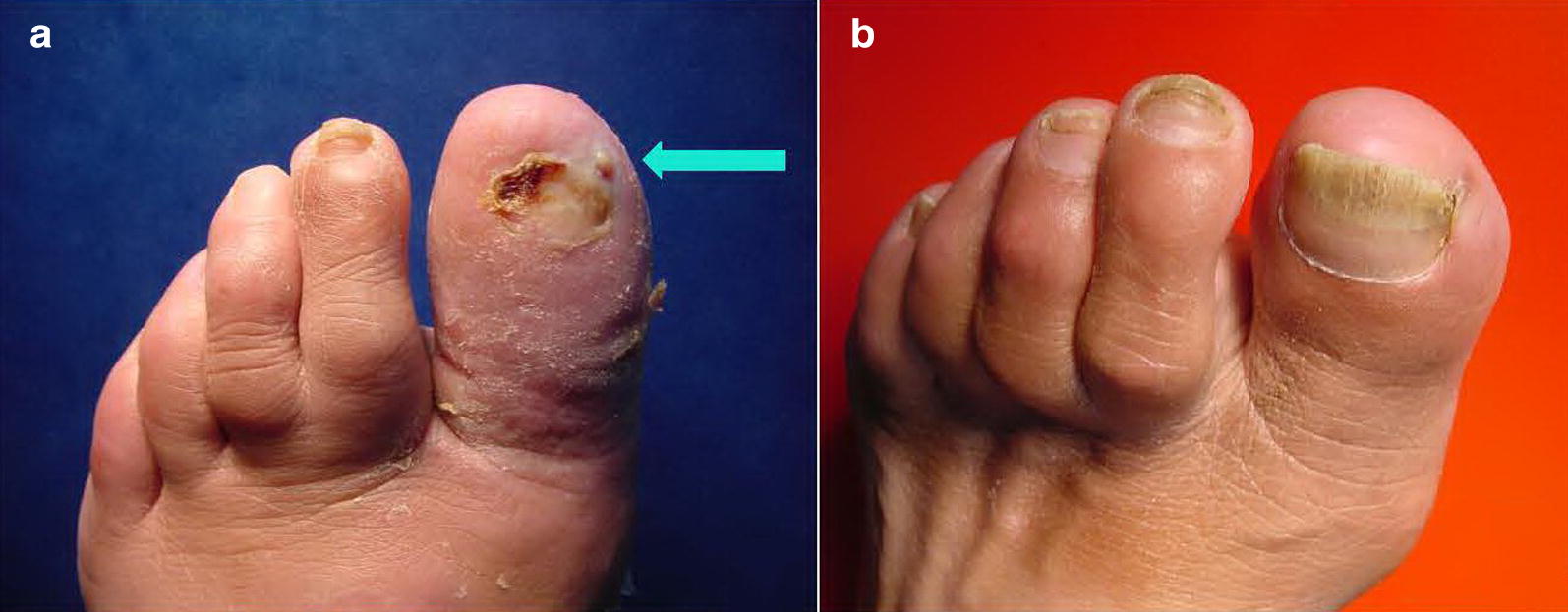
**a**, **b** Left foot of the patient. **a** 1st toe at initial visit (“sausage toe”). DFU (arrow). **b** Same toe 1 ½ year later

**Fig. 2:**
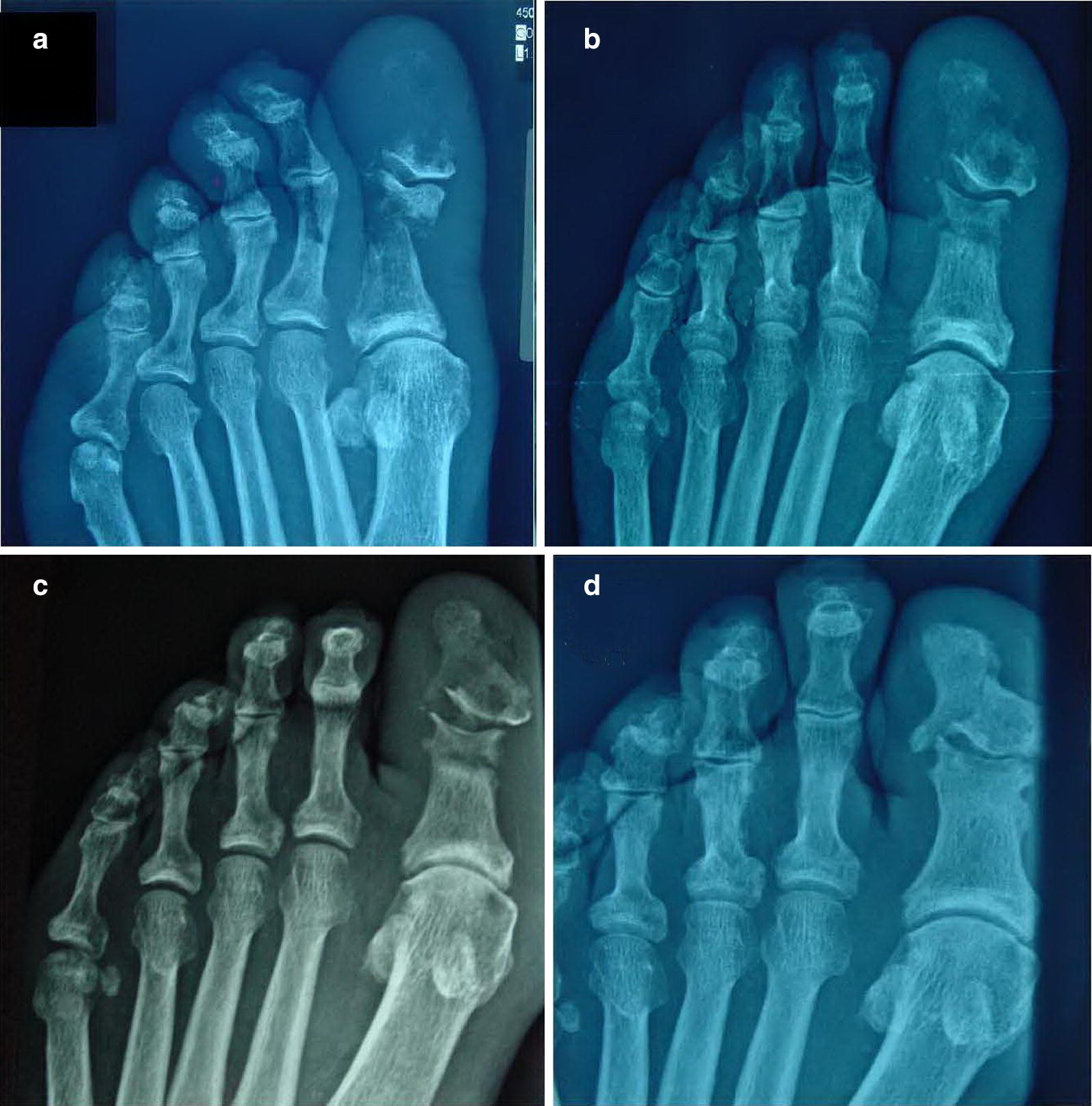
**a**–**d** Plain radiographs of the patient. Left foot. **a** 1st toe at initial visit. **b** Same toe 5 weeks later. **c** Same toe 7 weeks later. **d** Same toe 1 year later

A 68-year-old male, resident of Crete island, with history of type 2 DM (diagnosed 2 years before, with satisfactory glycaemic control, HbA1c = 6.5%) and intermittent claudication (Fontaine class IIb) was referred to our diabetic foot clinic due to a 1-month history of erythema, swelling, tenderness and local warmth of the left lower limb along with paronychia and the presence of a diabetic foot ulcer (DFU) in distal phalanx of 1st toe of the left foot probed to bone. The patient had been hospitalized for 10 days in a peripheral hospital, where he underwent surgical nail removal and surgical debridement, and was treated with double antibiotic therapy. He was prescribed antibiotics for 1 week after discharge. Upon admission to our hospital, he had typical “sausage toe” (1st toe of the left foot), Fig. 1a. Inflammation markers were significantly elevated (erythrocyte sedimentation rate (ESR) = 101 mm/h, C-reactive protein (CRP) = 16.3 mg/L (normal values: < 3.3 mg/L) and white blood cell count (WBC) = 13.310/μL. The rest of biochemical analysis was normal. X-ray of the left foot revealed destruction of proximal and distal phalanx of the 1st toe (image compatible to osteomyelitis), Fig. 2a. He was initially treated with intravenous daptomycin, aztreonam, plus low-molecular weight heparin and pentoxifylline. Swab culture after debridement showed methicillin-resistant *Staphylococcus aureus* (MRSA) and *Stenotrophomonas maltophilia*, both sensitive to ciprofloxacin, so antibiotic therapy was switched to ciprofloxacin and clindamycin. Nasal swab culture was also positive for MRSA, and nasal mupirocin was given.

Additionally, he underwent colour Doppler ultrasound and CT angiography of the lower extremity arteries. The patient presented 95% stenosis of the left popliteal artery and total occlusion of the posterior tibial artery of the right lower limb. Two weeks after admission, patient was discharged and continued his antibiotic treatment with ciprofloxacin and clindamycin per os as outpatient.

After discharge, the patient was seen on weekly basis. A gradual clinical improvement and a significant reduction of the inflammation marker levels were observed. Five weeks after discharge, the patient underwent a successful angioplasty, in order to perform revascularization and restore an adequate blood flow to his left lower limb. Blood tests at this time point were almost within normal ranges (ESR = 17 mm/h, CRP = 4.01 mg/L and WBC = 8.160/μL. He continued antibiotic treatment for 5 more weeks (a total of 3 months). After the end of treatment, inflammation markers had returned to normal (ESR = 19 mm/h, CRP < 3,3 mg/L and WBC = 7.800/μL). Plain X-ray showed focal osteogenesis at the damaged proximal and distal phalanx of the 1st toe. Clinically, the patient presented minimal edema of his toe without erythema and his DFU was almost healed (Fig. 2b, c).

Eventually 4 months after the end of the treatment, the patient presented with complete healing of his DFU, reconstruction of his osteomyelitis defects and complete restoration of his toe nail and his foot functionality. He was able to walk 3 km without claudication. One and a half year after his initial visit, the patient remains in good shape, and plain X-ray is almost normal (Figs. 1b, d).

## Discussion and conclusions

The management of osteomyelitis as a result of an infected DFU is very important. Diagnosis is difficult, and incorrect and postponed treatment can lead to amputation. Therefore, an early and accurate diagnosis of osteomyelitis is mandatory. This situation becomes more difficult when there are other comorbidities, such as peripheral artery disease, which causes ischaemia in an already infected lower limb. Additionally, resistance to antibiotics is increasing in diabetic population and it consists another problem that complicates treatment of osteomyelitis [3].

Nowadays there is an increasing tendency towards nonsurgical therapy of osteomyelitis in DF, which traditionally has been surgically treated [3–5]. According to IDSA guidelines [3], nonsurgical treatment can be tried if:

- There is no persisting sepsis.
- Patient can receive and tolerate appropriate antibiotic therapy.
- Degree of bony destruction has not caused irretrievable compromise to mechanics of foot.
- Patient prefers to avoid surgery.
- Patient comorbidities confer high risk to surgery.
- There are no contraindications to prolonged antibiotic therapy.
- Surgery is not otherwise required to deal with adjacent soft tissue infection or necrosis.

In the case of our patient, he fulfilled most of the above described criteria (no sepsis, tolerable antibiotic therapy, minor bony destruction, no contraindications to prolonged therapy, no necrosis).

The patient was a high-risk patient due to history of PAD. However, he avoided amputation and was successfully treated. He was hospitalized only for 2 weeks for intravenous antibiotics, while antimicrobial treatment lasted 3 months in total.

It is remarkable that the plain X-ray of the foot was nearly entirely restorated.

Furthermore, our patient benefited by a successful revascularization process (angioplasty). It is important to mention that his foot functionality was completely restored, since blood flow to his foot became normal and he did not present intermittent claudication anymore.

In conclusion, we present a case of a man with DM who was admitted with a typical bacterial osteomyelitis of the foot, caused by an infected diabetic foot ulcer. Despite his comorbidity of peripheral artery disease, he was nonsurgically treated successfully, and was discharged from hospital after an uneventful short stay.

## Abbreviations

DF: diabetic foot
DFU: diabetic foot ulcer
DM: diabetes mellitus
PAD: peripheral artery disease
